# Effects of Microorganisms on Growth Performance, Body Composition, Digestive Enzyme Activity, Intestinal Bacteria Flora and Antimicrobial Peptide (AMP) Content of Black Soldier Fly Larvae (*Hermetia illucens*)

**DOI:** 10.3390/ani13172722

**Published:** 2023-08-26

**Authors:** Yongqi Meng, Xiuxia Zhang, Zelong Zhang, Jiajun Li, Peihua Zheng, Juntao Li, Jiarui Xu, Jianan Xian, Yaopeng Lu

**Affiliations:** 1 Hainan Provincial Key Laboratory for Functional Components Research and Utilization of Marine Bio-Resources, Institute of Tropical Biosciences and Biotechnology, Chinese Academy of Tropical Agricultural Sciences, Haikou 571101, China; 2 Ocean College, Hainan University, Haikou 570228, China; 3 Key Laboratory for Biology and Genetic Resources of Tropical Crops of Hainan Province, Hainan Institute of Tropical Agricultural Resources, Haikou 571101, China

**Keywords:** *Hermetia illucens*, antimicrobial peptide, growth performance, digestibility, intestinal microbe

## Abstract

**Simple Summary:**

Antimicrobial peptides (AMPs) are a class of small-molecule active polypeptides. Because of their good antibacterial activity, stability and safety, AMPs are considered one of the potential alternatives to antibiotics. Black soldier fly (*Hermetia illucens*) is an excellent material for the study of AMPs. This study aims to screen optimum microbial strains and concentration to support the growth performance and AMP induction of *H. illucens*. Six microorganisms were selected as feed additives for *H. illucens* by tracking the growth performance, proximate composition, digestive ability and AMP content in the first trial. Microorganism efficiency screening results showed that *Rhodopseudomonas palustris* (RP) could improve growth performance, digestive ability and AMP content of *H. illucens*. Therefore, RP was selected to prepare the diets and was incorporated into diets for *H. illucens* at different levels. The results showed that RP is superior to the other strains as a feed additive for the *H. illucens* larvae, and we recommend the addition of 1.22 × 10^9^–1.22 × 10^10^ CFU/g RP to promote the growth and AMP content of *H. illucens*. This study provides new ideas for large-scale production of AMPs and lays the foundation for the development of AMPs as feed additives to substitute antibiotics.

**Abstract:**

Escherichia coli (EC), Staphylococcus aureus (SA), Bacillus subtilis (BS), Rhodopseudomonas palustris (RP), Saccharomyces cerevisiae (SC) and Lactobacillus plantarum (LP) were selected as feed additives for black soldier fly (Hermetia illucens) by tracking the growth performance, proximate composition, digestive ability and antibacterial peptides (AMPs) content in the first trial. Microorganism efficiency screening results showed that RP could improve growth performance, digestive ability and AMP content of H. illucens. Therefore, RP was selected to prepare the diets and was incorporated into diets for H. illucens at levels of 0 (R0), 1.22 × 10^6^ (R1), 1.22 × 10^7^ (R2), 1.22 × 10^8^ (R3), 1.22 × 10^9^ (R4) and 1.22 × 10^10^ (R5) CFU/g. After 5 d of feeding, larvae fed the R2-R5 diets had higher weight gain and specific growth rates. Different concentrations of RP had no significant effect on larval body composition. R4–R5 could improve the digestibility and expression of AMPs in larvae. Moreover, RP could significantly increase the abundance of Lactobacillus and Rhodopseudomonas and decrease the abundance of Proteus and Corynebacterium. Therefore, RP is superior to the other strains as a feed additive for H. illucens larvae, and we recommend the addition of 1.22 × 10^9^–1.22 × 10^10^ CFU/g RP to promote the growth and AMP content of H. illucens.

## 1. Introduction

The large-scale and intensive development of aquaculture has caused many farming diseases, and antibiotics have been widely used to prevent and control bacterial diseases [1,2,3]. However, the misuse of antibiotics can result in many problems in aquatic animals, such as the emergence of drug-resistant bacteria, drug residues, microecological imbalances and immune system disruption [4,5]. Multidrug resistant bacterial infections caused by antibiotics threaten people’s life and health safety [4]. Many countries around the world have issued laws banning the use of antibiotics [6]. Therefore, finding new efficient and environmentally friendly feed additives to replace antibiotics has become an urgent task for research and development.

Antimicrobial peptides (AMPs) are a class of small-molecule active polypeptides. Because of their good antibacterial activity, stability and safety, AMPs are considered one of the potential alternatives to antibiotics [7]. AMPs have broad-spectrum resistance to bacteria, fungi, viruses and parasites [8,9]. In the immune system, AMPs are important effectors and modulators that perform a variety of physiological functions, including the recruitment of immune cells, release of chemokines and regulation of pro-inflammatory responses [10,11]. Antimicrobial peptides have the advantages of good stability, natural source and no pollution, which is a good alternative to antimicrobials [12]. However, there are still many problems in the application of AMPs in aquaculture. There are many kinds of AMPs, but the expression of the antimicrobial mechanism is not clear. To apply AMPs to aquatic animal feed, we should understand the relationship between the function and structure of AMPs, and a suitable one that should be selected for different species. In addition, the AMO production cost is high. The extraction process is complex. The yield is limited and cannot be used on a large scale in animal breeding. The promotion of AMPs depends on their efficient production and cost reduction.

Black soldier fly (*Hermetia illucens*) can transform organic waste into usable biological resources. Its nutritional value is high. The content of protein, lipid and trace elements in the body accounts for 35–64% of the dry weight of the larvae and has good application in feed protein replacement [13,14]. *Hermetia illucens* contains more than 50 AMP genes, and AMPs produced in its body have a highly effective antibacterial ability [15]. *Hermetia illucens* is an excellent material for the study of AMPs [16]. There are several methods for the production of AMPs from *H. illucens*, such as the molecular technology method [17,18], the hemolymph collection method [19] and the grinding and extraction method [20]. The AMP content in the body of *H. illucens* is low under normal conditions. Microbial needling infection is a relatively common method to induce the expression of AMPs in *H. illucens.* Park et al. reported that using *Staphylococcus aureus* needling could induce the production of AMPs in *H. illucens* [21]. Lee et al. reported that using *Lactobacillus casei* with acupuncture could induce the production of AMPs in *H. illucens* [22]. Similarly, the expression of AMPs can be induced by *Escherichia coli* needling [19]. Bacterial injection and other methods are commonly used to stimulate the production of AMPs in *H. illucens* [21,22]. However, these methods are not applicable to the production of AMPs and are not realistic in production practice.

Therefore, this study aimed to screen optimum microbial strains and concentration to support the growth performance and AMP induction of *H. illucens*. The microbial strain efficiencies of growth enhancement and AMP induction improvement for *H. illucens* of six microbial strains were evaluated. The optimal microbial strains of feed at different concentrations were prepared, and their mechanism of influence was evaluated by tracking the growth performance, intestinal tract bacteria and AMPs induction.

## 2. Materials and Methods

### 2.1. Microbial Culture

Six microorganisms, including *E. coli* (EC), *S. aureus* (SA), *Bacillus subtilis* (BS), *Rhodopseudomonas palustris* (RP), *Saccharomyces cerevisiae* (SC) and *Lactobacillus plantarum* (LP) were used in this study. EC, SA and BS were cultured in LB (Luria–Bertani) (Huankai Biology, Guangzhou, China) media at 37 °C for 12 h. RP was incubated in media at 30 °C for over 96 h under 3000 lx light intensity. SC was cultured in YPD (yeast extract peptone dextrose) (Huankai Biology, Guangzhou, China) media at 30 °C for 12 h. LP was incubated in a stationary state in MRS (deMan, Rogosa and Sharpe) (Huankai Biology, Guangzhou, China) media at 37 °C for 12 h. After cultivation, bacteria were collected by centrifugation at 3000× *g* for 10 min and resuspended in phosphate buffer solution (PBS) to prepare experimental diets.

### 2.2. Experimental Diet Preparation

The basic feed of black soldier fly larvae were made based on a previous study [23]. The diet consists of 50% wheat bran, 30% soya bean meal and 20% corn meal. Distilled water was added to make the water content of the feed 55%. The proximate composition of the experimental diet is shown in Table 1. Two batches of the experimental diet were prepared in this study. For the first batch of diet preparation, EC, SA, BS, RP, SC and LP were individually added to the experimental diets at the level of 1.22 × 10^8^ colony forming unit (CFU)/g. The diet supplemented with PBS was used as the control diet (control) for the first trial. For the second experimental diet preparation, RP was incorporated into experimental diets at levels of 0 (R0), 1.22 × 10^6^ (R1), 1.22 × 10^7^ (R2), 1.22 × 10^8^ (R3), 1.22 × 10^9^ (R4) and 1.22 × 10^10^ (R5) CFU/g. The diet supplemented with PBS was used as the control diet (R0). In the experiment, the strains were resuspended in equal doses of PBS added to the feeds. To ensure the safety of the experiment, pathogenic bacteria (EC, SA and BS) were added to the feed only after being inactivated.

### 2.3. Hermetia illucens Acclimation

*Hermetia illucens* eggs were bought from a private farm in Guangzhou, Guangdong, China. Eggs in plastic bottles with corn flour were transported to the laboratory at the Institute of Biology, Chinese Academy of Tropical Agricultural Sciences, Haikou, Hainan, China. During acclimation, eggs were reared for 3 d in the storage box (60 cm × 45 cm × 30 cm) at 28 °C and 55% relative humidity. When hatching, eggs were put in a cool place to avoid direct sunlight and rapid water evaporation.

### 2.4. Experimental Design and H. illucens Rearing

Two experiments were conducted in this study. In the first experiment, optimal microorganisms were screened for growth and induction of AMPs expression in *H. illucens*. Seven experimental diets containing a single bacterium (Control (supplement with PBS), EC, SA, BS, RP, SC and LP) were offered to *H. illucens* larvae (initial weight of 0.01 ± 0.002 g) for 5 d. After 5 d of feeding, growth performance and AMP concentration were determined. Because the RP improved the growth performance and AMP concentration of the *H. illucens* larvae in the first trial, RP was used to prepare the diet, and the optimal concentration of the RP was evaluated in the second trial. In the second trial, larvae with an initial mean weight of 0.01 ± 0.002 g were assayed for growth performance, proximate composition, including ash, crude protein and crude lipid, and digestive ability, including amylase, lipase and trypsin activities in the gut and intestinal microbiota, and AMP concentration after 5 d of feeding with the RP diets (R0 (supplement with PBS), R1, R2, R3, R4 and R5).

During the period of experiments, healthy pretreated larvae of the same size were put into feeding boxes (ladder PP plastic boxes with top bottom 21.5 cm × 14.5 cm, bottom 17.5 cm × 10.5 cm and height 6.9 cm). Feed (45 g) and bacteria solution (55 mL) are added to each box of the experimental group and mixed well. For the first experiment, the concentration of each bacterial solution was 1 × 10^8^ CFU/mL. For the second experiment, the concentration of the bacterial solution in each RP group was 1 × 10^6^ (R1), 1 × 10^7^ (R2), 1 × 10^8^ (R3), 1 × 10^9^ (R4) and 1 × 10^10^ (R5) CFU/mL. Each treatment group was set up with 6 replicates, and 100 larvae were in each feeding box. The pretreated feed was added to the feeding box and placed in a biochemical incubator (temperature 28 °C, relative humidity 55%) for feeding. The experimental feed was spread evenly on the bottom of the box, and the black soldier fly larvae were kept on the feed. The feed was given sufficiently during the experiment, and the water content was relatively stable.

### 2.5. Sample Collection

Each group of *H. illucens* was rinsed with water and sterilized with 75% alcohol, after which, *H. illucens* were collected and frozen in liquid nitrogen after the breeding experiment. The intact black soldier fly larvae were stored at −80 °C for further analysis.

### 2.6. Growth Performance

After a 5 d feeding trial, 20 larvae were chosen randomly from every replication to calculate weight gain rate (WGR) and specific growth rate (SGR). The following methods by Zokaeifar et al. [24] and Yeh et al. [25] were used:

WGR (%) = [(Final weight − Initial weight)/Initial weight] × 100

SGR (% day^−1^) = [(ln Final weight − ln Initial weight)/Days] × 100.

### 2.7. Proximate Composition

The crude protein, crude lipid and ash of whole larvae were determined according to the Association of Official Analytical Chemists (AOAC) (1995) [26]. Crude protein, crude lipid and ash were determined using K8400 Kjeltec Analyzer (Fossana Lyticab, Shanghai, China), Soxtec System HT (SE-A6, Alvah, Shanghai, China) and muffle furnace, respectively.

### 2.8. Digestive Ability

Whole larvae were used to determine the digestive ability of *H. illucens*. Amylase, lipase and trypsin activities were determined using their respective kits (catalog no. C016-1-1, A054-2-1 and A080-2) following the manufacturer’s instructions (Nanjing Jiancheng Bioengineering Institute, Nanjing, China).

### 2.9. Intestinal Microbiota

Three mixed samples per group were used in performing the intestinal flora analysis. Total bacterial DNA was extracted from the peeled larvae using Tguide S96 DNA Kit (Tiangen, Beijing, China) according to the manufacturer’s instructions. The quality and concentration of DNA were measured using a NanoDrop One spectrophotometer (Thermo, Shanghai, China). The V4 region of the bacterial 16S rRNA gene was amplified by PCR using the primers, including 515F (5′-GTGYCAGCMGCCGCGGTAA-3′) and 806R (5′-GGACTACNVGGGTWTCTAAT-3′). PCR was performed in a 10 μL reaction system containing 5 μL PCR buffer (KOD FX Neo buffer, Toyobo, Osaka, Japan), 1 μL DNA template, 2 μL dNTP, 0.2 μL KOD FX Neo, 0.3 μL of forward primer (10 μM), 0.3 μL of reverse primer (10 μM) and 1.2 μL ddH_2_O. The PCR conditions were as follows: 95 °C for 5 min, followed by 25 cycles of 95 °C for 30 s, 50 °C for 30 s and 72 °C for 40 s and finally 72 °C for 7 min. The purified PCR products were sequenced on the Illumina novaseq6000 platform (Illumina, San Diego, CA, USA) to generate paired-end sequences. According to quality of single nucleotide, raw data were primarily filtered by Trimmomatic [27] (version 0.33). Identification and removal of primer sequences were performed using Cutadapt [28] (version 1.9.1). PE reads obtained from previous steps were assembled by USEARCH [29] (version 10) and followed by chimera removal using UCHIME [30] (version 8.1). The high-quality reads generated from the above steps were used in the following analysis. OUT, diversity, rarefaction curve and abundance were all performed by Beijing Biomarker Technologies (Beijing, China).

### 2.10. Extraction and Determination of AMPs

The lyophilized larvae were well-ground and extracted with acidified methanol (methanol/water/acetic acid; 90/9/1) [31]. Extracts were centrifuged at 1600× *g* for 10 min at 4 °C and dried in a rotary evaporator under reduced pressure. Proteins and lipids were removed from the samples by sequential extraction with chloroform and ethyl acetate. All fractions were lyophilized and stored at −20 °C for further use. The concentration of AMPs was determined using kits following the manufacturer’s instructions [32] (Nanjing Jiancheng Bioengineering Institute, Nanjing, China).

### 2.11. Antibacterial Effect

Using the filter paper diffusion method [32], the extract was set as the negative control (NC) and 120 μg/mL of florfenicol was set as the positive control (PC). In addition, 20 μL of *E. coli* at 1 × 10^8^ CFU/mL was evenly coated in the plates containing LB medium. Then, filter paper of 6 mm in diameter was sterilized and soaked in the crude extract of AMPs in the control group and each experimental group for 2–5 min and taken out with sterile forceps for 5 min at room temperature into drug-sensitive paper sheets. The distance between each paper sheet on the culture medium should be greater than 24 mm. The distance between the paper sheet and the inner edge of the Petri dish should be greater than 15 mm.

### 2.12. Statistical Analysis

All experimental data are presented as means ± standard deviation (SD). The data were analyzed for significance by one-way analysis of variance (ANOVA). When differences were found among groups by one-way ANOVA, Tukey’s multiple-comparison test (SAS Institute, Cary, NC, USA) was conducted to examine differences among the controls and treatments. Statistical analyses were performed with Statistical Package for the Social Science version 18 (SPSS 18.0) software with *p* < 0.05 as statistically significant.

## 3. Results

### 3.1. First Trial: Screening Possible Microbial Strain Candidates

#### 3.1.1. Growth Performance and Survival Rate

During 5 d of the growth trial, the larvae did not die. Growth parameters of *H. illucens* larvae are presented in Table 2. RP, SC and SA groups exhibited significantly higher WGR and SGR than the control group (*p* < 0.05).

#### 3.1.2. Proximate Composition

The effect of dietary microbiota on the proximate composition (Crude protein, crude lipid and ash) of *H. illucens* is presented in Table 3. All treatments showed no significant differences in crude protein content. SC group exhibited a significant increase (*p* < 0.05) in crude lipid and ash content compared to the other groups.

#### 3.1.3. Digestive Ability

Microbial strain supplementation of *H. illucens* larvae affected the activity of α-amylase, lipase and trypsin in the gastrointestinal tract of *H. illucens* larvae (Table 4). The *H. illucens* larvae fed RP had the highest α-amylase, lipase and trypsin activities. The α-amylase activity of RP and SC groups was significantly enhanced (*p* < 0.05) compared to that of the other groups. *H. illucens* larvae fed with the RP, SC and SA had increased lipase activity significantly higher than that of the other groups (*p* < 0.05). Trypsin activity was significantly higher in the RP, BS, SA and SC groups when compared to that of the other groups (*p* < 0.05).

#### 3.1.4. AMPs Content

The AMP content of each group of *H. illucens* larvae was measured after feeding diets were mixed with EC, SA, BS, RP, SC and LP (Table 5). The AMP content of *H. illucens* in EC, SA, RP and SC groups was significantly higher than that in the control group, with the RP group having the most significant AMP content (*p* < 0.05).

#### 3.1.5. Antibacterial Effect

The diameter of the inhibition circles of each feed group, NC and PC, are shown in Table 6. The corresponding inhibition pictures are shown in Figure S1. The diameter of the inhibition circle of the NC group was 0. The diameter of the inhibition circle of the PC group was greater than that of all experimental groups of the AMPs in *H. illucens* (*p* ˂ 0.05). The diameter of the inhibition circle of the RP group was significantly greater than that of the BS, LP and control groups (*p* ˂ 0.05); the diameter of the EC, SA, RP and SC groups was not significantly different (*p* > 0.05).

### 3.2. Second Trial: The Evaluation of Efficiencies of Different Concentrations of the Microbial Strain

#### 3.2.1. Growth Performance and Survival Rate

To evaluate the effect of RP on the growth performance and survival rate of *H. illucens*, we prepared diets containing five different concentrations of RP, R1 (1.22 × 10^6^ CFU/g), R2 (1.22 × 10^7^ CFU/g), R3 (1.22 × 10^8^ CFU/g), R4 (1.22 × 10^9^ CFU/g) and R5 (1.22 × 10^10^ CFU/g), to feed *H. illucens*. After a 5 d feeding period, the larvae did not die and WGR and SGR were measured. Supplementation with the RP tested improved growth performance compared to the control group (Table 7). In addition, R4 and R5 groups displayed significantly higher WGR and SGR than the R0 group (*p* < 0.05).

#### 3.2.2. Proximate Composition

The whole-body compositions are shown in Table 8. R4 and R5 promoted the crude lipid content of the body compositions of *H. illucens* larvae compared to groups R0, R1, R2 and R3. There were no significant differences in ash content between the five treatments and the control group (*p* > 0.05).

#### 3.2.3. Digestive Ability

The activities of α-amylase, lipase and trypsin in larvae fed with RP are presented in Table 9. The activities of α-amylase, lipase and trypsin in the intestinal tract of *H. illucens* after feeding with a diet containing RP were significantly increased (*p* < 0.05).

#### 3.2.4. Antibacterial Peptide Content

After feeding diets mixed with different concentrations of RP, the AMP content of each group of *H. illucens* larvae was measured (Table 10). As the concentration of RP increased, the AMP content of *H. illucens* increased significantly (*p* ˂ 0.05). The AMP content of *H. illucens* in R1, R2, R3, R4 and R5 groups was significantly higher than that in R0, with the most significant AMP content in groups R4 and R5 (*p* ˂ 0.05).

#### 3.2.5. Intestinal Microbiota

**Figure 1:**
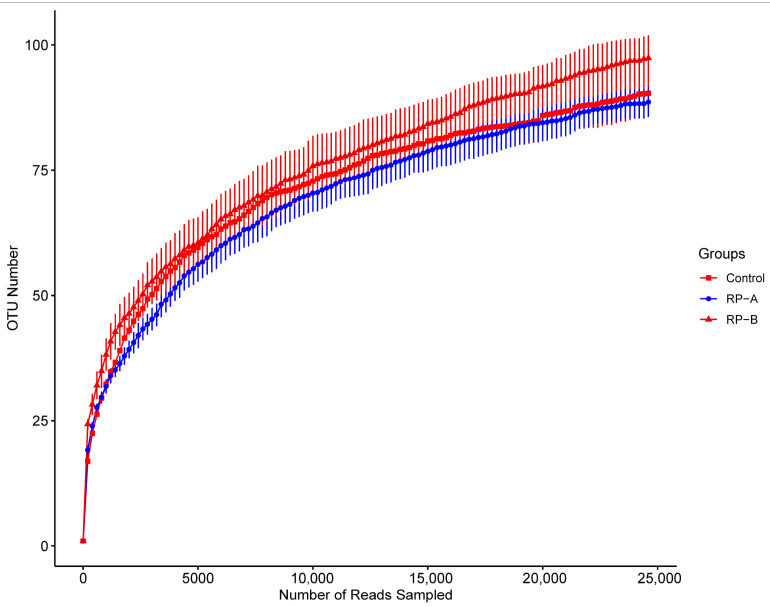
Rarefaction curves of samples.

Using 16S rDNA high-throughput sequencing, we measured the microbiota composition in the *H. illucens* intestine after feeding with a diet containing R0 (Control), R1 (RP-A) and R5 (RP-B). Raw sequencing information is shown in Table S1. Sangon Biotech sequencing of intestinal microbes produced 446,670 sequences from nine samples. Using cluster analysis of valid sequences according to 97% similarity, a total of 141 operational taxonomic units (OTUs) were obtained. The dilution curve tended to approach the saturation plateau, which indicates that most of the microorganisms have been detected in this study, and the results are representative (Figure 1).

**Figure 2:**
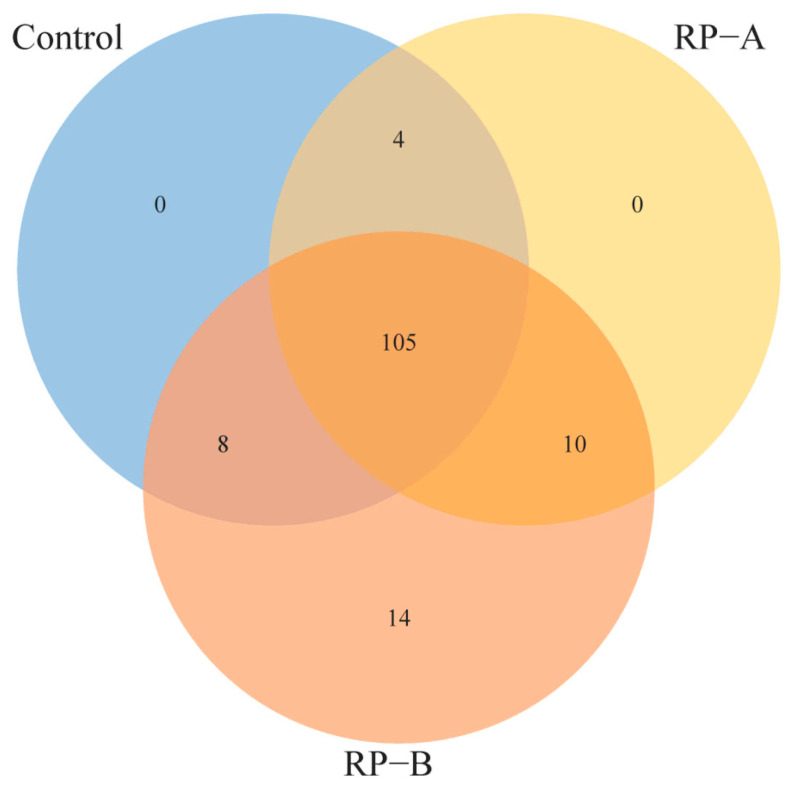
Venn diagram analysis of species.

Venn diagram showed that 105 OTUs were shared among the three groups, and 117, 119 and 137 core OTUs were identified in the control, RP-A and RP-B groups, respectively. The DNA sequences from samples of the RP-B group had the most abundant unique OTUs (14) (Figure 2).

**Figure 3:**
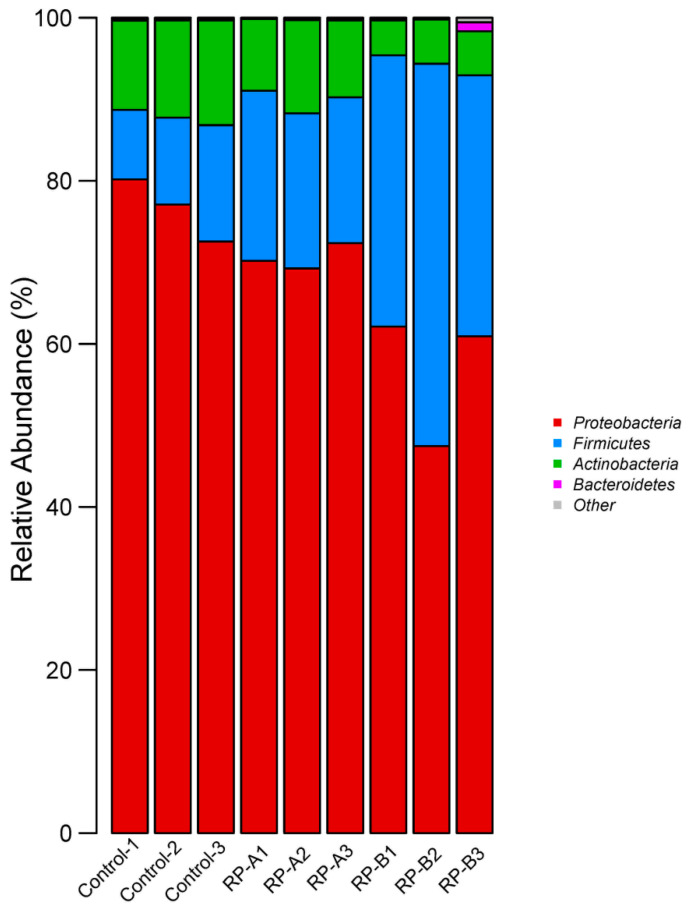
The percentage of community abundance on phylum level. The phylum with less than 1% relative abundance was collected to other, other = 30.

The composition of intestinal microbes from the three groups at the phylum level was mainly *Proteobacteria*, *Firmicutes* and *Actinobacteria* (Figure 3). However, the relative proportion in each group differed. *Proteobacteria* was the dominant phylum in the three groups, accounting for 76.60%, 70.59% and 56.85%, respectively. Interestingly, the relative abundance of *Firmicutes* in the RP-B group (37.39%) increased compared with that in the control group (11.16%). The relative abundance of *Actinobacteria* in the RP-B group (5.03%) decreased compared with that in the control group (11.91%).

**Figure 4:**
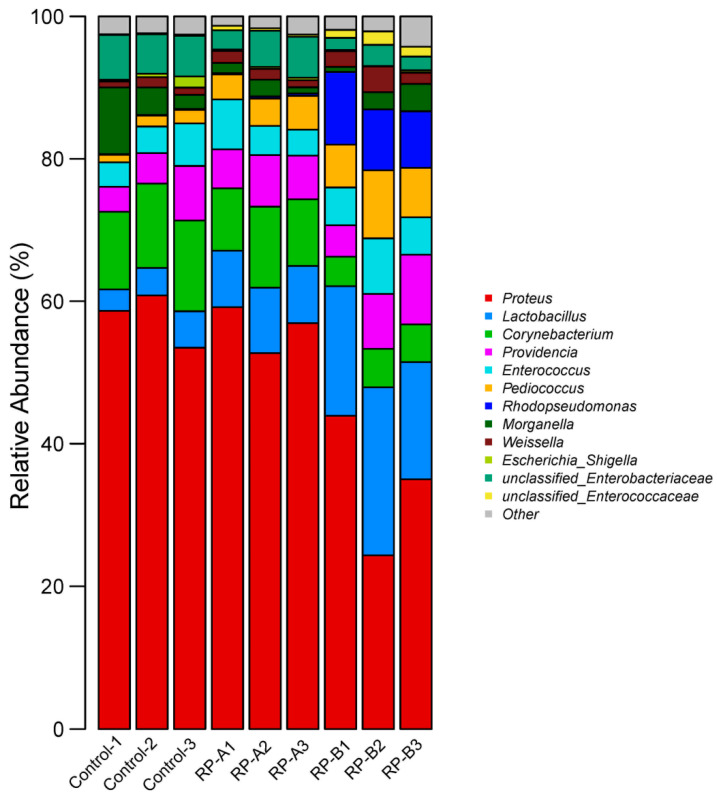
The percentage of community abundance on genus level. Note: The genus with relative abundance less than 1% was collected to other, other = 30.

At the level of genus classification (Figure 4), *Proteus* was dominant in the three groups, and the average relative abundance of *Proteus* in the RP-B group was 34.41%. Still, the relative abundance was significantly different between the control (57.64%) and RP-A groups (56.26%) (*p* < 0.05). The genera of *Corynebacterium* (11.84%) and *Providencia* (5.16%) were dominant in the control group. *Corynebacterium* (9.84%) and *Lactobacillus* (8.39%) were dominant in the RP-A group. *Lactobacillus* (19.42%) and *Rhodopseudomonas* (8.90%) were dominant in the RP-B group.

Moreover, the Shannon and Ace indexes of the intestinal microorganism community of the RP-B group were significantly higher than those of the control and RP-A groups. Regarding the Chao index, the RP-B group had the same index as the control and RP-A groups; no significant differences were observed. The Simpson index was significantly lower than that of the control and RP-A groups (Table 11).

**Figure 5:**
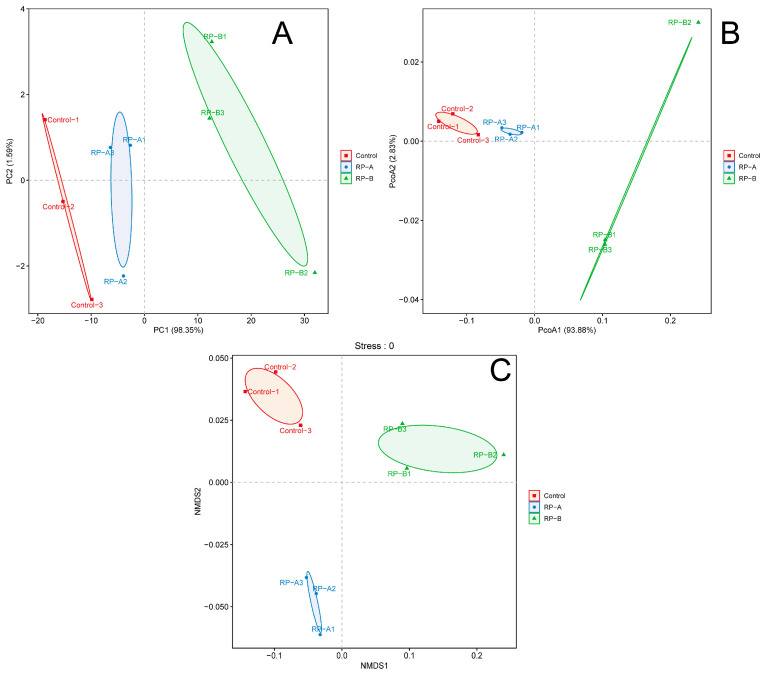
Differences in bacterial beta diversity between the treatment groups and RP group. (**A**) Principal coordinate analysis (PCA), (**B**) two-dimensional principal coordinate analysis (PCoA), (**C**) nonmetric multidimensional scaling (NMDS) analysis.

The microbial communities of each group appeared as one major cluster along PCA2 and along PCA1 (Figure 5). The principal coordinate (PCoA) cluster analysis indicated a separation between the control and RP groups. NDMS analyses revealed a clear separation of the three groups.

**Figure 6:**
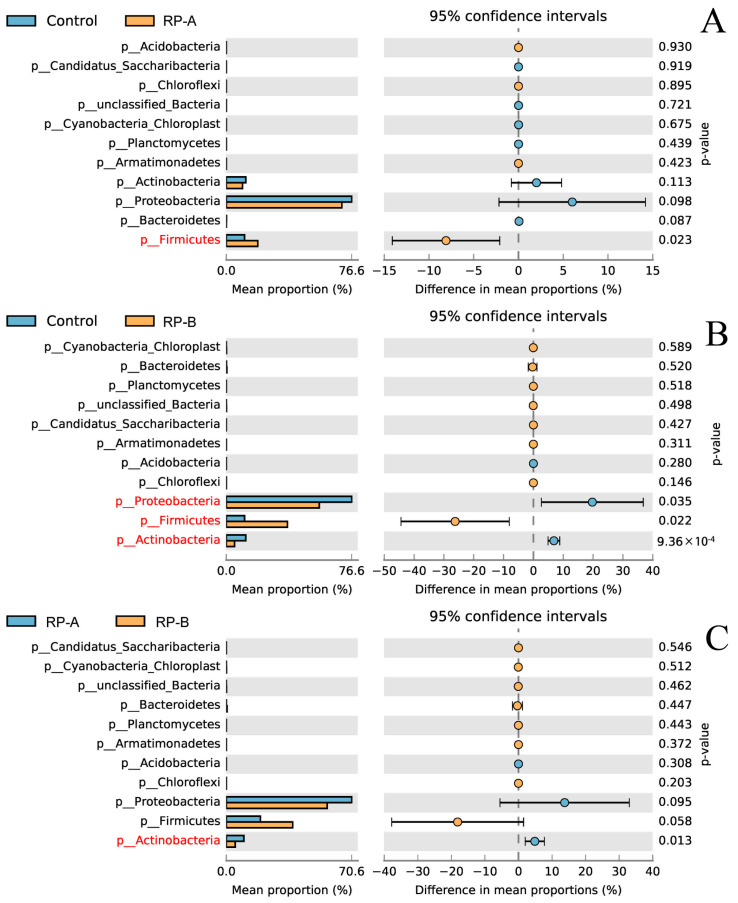
Student’s *t*-test bar plot on phylum level between control and RP-A and RP-B groups. (**A**) Analysis of differences on phylum level bewteen Control groupand RP-A group, (**B**) Analysis of differences on phylum level bewteen Control groupand RP-B group, (**C**) Analysis of differences on phylum level bewteen RP-A groupand RP-B group. Phylums with a *p*-value less than 0.05 are marked red.

Welch’s *t*-test was performed to evaluate the differentially abundant genera. RP-A and RP-B groups showed a significantly higher abundance of *Lactobacillus* and *Pediococcus* than the control group (Figure 6A). The RP-B group presented a significantly higher abundance of *Rhodopseudomonas* and a lower abundance of *Corynebacterium* and *Proteus* (Figure 6B,C). The RP-B group displayed a significantly higher abundance of *Rhodopseudomonas, Lactobacillus* and *Clostridium* and a lower abundance of *Corynebacterium* and *Proteus* than the RP-A group.

## 4. Discussion

Insect meal is a good substitute for fish meal in aquatic feed because of its high protein content and rich nutrition [33]. Insect AMPs have good antibacterial and immunomodulatory activities and are a good substitute for antibiotics [17]. Insects can turn waste into biological resources. They can reproduce on a large scale, in line with the requirements of modern health, environmental protection and sustainable development [34]. The production of AMPs using *H. illucens* has the advantages of environmental protection, high yield and low cost.

### 4.1. Effect of Feed Supplementation with Different Strains on H. illucens Larvae

#### 4.1.1. Effect of Feed Supplementation with Different Strains on the Growth and Development of *H. illucens* Larvae

The impact of microbiota on host development, metabolism and immunology has made microbial research an integral part of organismal biology. The addition of microorganisms to feed may affect the growth and development of animals [35]. This study determined the growth performance and body composition of *H. illucens*-fed diets containing different strains. Adding microorganisms to feeds can promote animal growth and development [35,36,37]. Lee et al. [38] reported that adding *B. subtilis* to feed improved growth performance and nutrient digestibility in weaned piglets. Merrifield et al. [39] reported that feed supplementation with probiotics could improve feed conversion, specific growth rate and protein efficiency in rainbow trout (*Oncorhynchus mykiss*). Kannan et al. reported that *S. cerevisiae* contributes to altered metabolic pathways in the *H. illucens* larvae and enhances larval body weight [40]. This study showed that feed addition of inactivated SA, RP and SC significantly improved the growth performance of *H. illucens* larvae. However, the addition of EC, BS and LP did not significantly affect the growth performance of *H. illucens* larvae. The large variation in the effect of different strains on the growth of *H. illucens* may be due to the different modes of action between the strains and the microorganisms in the *H. illucens* or because of the direct effect the development of the host. Studies reported that feed supplementation with microorganisms failed to promote the growth of the host. Hidalgo et al. [41] reported that feed supplementation with probiotics could not improve the growth performance of juvenile dentex (*Dentex dentex*), which indicates that the growth promoting effect of different strains on animals varies [42]. In the present study, the growth performance of *H. illucens* was significantly improved by the addition of SA, RP and SC to the feed, which implies a greater advantage of the application of these microorganisms in the feed of *H. illucens*. The *H. illucens* with feed supplementation of SA, RP and SC groups also had higher digestive enzyme activity, which can be explained by the fact that higher digestive enzyme activity implies higher digestive capacity and growth rate. The effect of probiotics on growth improvement was positively correlated with digestive enzyme activity and has been confirmed in many animal studies, such as in gilthead bream (*Sparus aurata)* [43], tilapia (*Oreochromis niloticus*) [44] and pacific white shrimp (*Litopenaeus vannamei*) [45], which may be attributed to the production of extracellular enzymes by the microflora after successful adhesion and colonization of the organism, which improves digestion [46]. This promotes the growth of the host.

#### 4.1.2. Effect of Feed Supplementation with Different Strains on the Body Composition of *H. illucens*

Feeding SC significantly increased the crude lipid and ash content of the *H. illucens* but had no significant effect on crude protein. Yu et al. [47] showed that feed supplementation with probiotics did not affect the body composition of *L. vannamei*. Seenivasan et al. reported that feed supplementation with yeast, *Bacillus* and *Lactobacillus* significantly increased the crude protein, crude lipid and ash content of freshwater prawn (*Macrobrachium rosenbergii*) [48]. Microbial effects on host components vary across species studies. The mechanisms of microbial effects on host components are unclear. In this study, only SC increased the crude lipid and ash content of the *H. illucens*, which could be due to SC affecting the metabolism of lipids in *H. illucens* and increasing lipid deposition. In addition, the changes in crude lipid and ash may be due to altered digestion and absorption of the feed, which may be a function of bioactive components (vitamins and amino acids) in the SC that facilitate the absorption of nutrients in the digestive tract. Ovie et al. reported that dietary yeast increased the crude lipid content and decreased the ash content of fingerlings (*Heterobranchus longifilis*) [49]. However, Darafsh et al. reported no significant effect of dietary yeast on crude lipid and ash content of Persian sturgeon (*Acipenser persicus*) [50]. These studies differed from the results of the present study, suggesting that there are differences in the mechanisms by which strains affect the body composition of different species. The effects of different microbe on the host vary and the exact mechanism remains to be explored.

#### 4.1.3. Induction of AMPs by Feed Supplementation with Different Strains

Insects have adapted to different habitats because their innate immune system provides them with a range of cellular and humoral responses against microorganisms [51]. In recent decades, significant advances have been made in the theory of immune defense due to the wide diversity of insects. They are an excellent source of biologically active molecules [52]. Insect AMPs are acquired immune factors, which can be induced, and have antibacterial, antidisease and antitumor properties [53]. Bacteria can induce the expression of host defense peptides in the organism, which improves the immune function of the organism [54].

Feeding diets containing different strains can induce the production of AMPs in yellow mealworm (*Tenebrio molitor*) larvae [55]. In this study, the addition of EC, SA, RP and SC to the diet-induced the production of AMPs in *H. illucens*, with RP, in particular, having the best inhibition effect. Choi et al. reported that *E. coli* could induce AMPs in *H. illucens*. AMPs had strong inhibition activity against Gram-negative bacteria, demonstrating the feasibility of feeding bacteria to induce AMPs in *H. illucens* [56]. The induction of AMPs by different bacteria had no significant effect on the overall antimicrobial activity of the bacteria. The immune response of *H. illucens* is nonspecific, and different induction sources can induce the insect AMPs to produce a peptide with a broad spectrum of antimicrobial activity and are not specific to a particular antigen-inducing substance [57,58]. The AMPs of *H. illucens* have a broad spectrum of antimicrobial activity and are promising agents in aquaculture.

### 4.2. Effect of Feed Supplementation with Different Concentrations of RP on H. illucens Larvae

#### 4.2.1. Effect of Feed Supplementation with Different Concentrations of RP on the Growth Performance of *H. illucens*

The first experiment showed that 1.22 × 10^8^ CFU/g of RP significantly improved the digestive capacity and growth of *H. illucens*. The second experiment explored the effect of different concentrations of RP on the growth and digestibility of *H. illucens* to arrive at the optimum growth-promoting concentration of RP. RP can provide protein, lipids, essential amino acids, essential fatty acids, vitamins and carotenoids [59,60,61,62,63], which can be used as a substitute for feed ingredients [64]. Feeding RP increases the growth and survival of fairy shrimp (*Streptocephalus sirindhornae*) [65]. Saejung et al. reported that three types of anaerobic RP increased the growth and survival of *S. sirindhornae* [66]. RP is rich in essential amino acids and polyunsaturated fatty acids (PUFA), which are extremely important for the growth of crustaceans [60,67]. Our results demonstrate that RP can promote the growth of *H. illucens*, similar to how they promote the growth of rotifers [68].

#### 4.2.2. Effect of Feed Supplementation with Different Concentrations of RP on the Body Composition of *H. illucens*

This study showed that adding RP to the feed did not affect the crude protein and ash content of *H. illucens*, but high concentrations of RP increased the deposition of lipids. RP is rich in amino acids and fatty acids, and high concentrations of RP provide more nutrients for *H. illucens* feed nutrition, which may alter the lipid metabolism and lead to higher deposition of lipids in *H. illucens*. RP contains a variety of B vitamins and pantothenic acid, which can be used as coenzymes to participate in the body’s metabolism and promote the animal’s growth and development [66]. These components can promote the metabolism of *H. illucens* and increase the deposition of lipids in the body.

#### 4.2.3. Induction of AMPs by Feed Supplementation with Different Concentrations of RP

Based on the first experiment, we demonstrated that RP had a better induction effect on the AMPs of *H. illucens*, and the second experiment explored the induction effect of different concentrations of RP on the AMPs. The results showed that higher concentrations of RP could better induce the expression of AMPs in *H. illucens*. AMPs were induced in *H. illucens* by needling injection and injection of bacterial solution [69]. In this study, feed supplementation with RP induced AMP production in *H. illucens*, which is methodologically simpler than by the needling and injection methods and suitable for large-scale AMP production. The ability of RP to induce the production of AMPs in *H. illucens* may be attributed to the fact that the metabolic substances produced by RP stimulate *H. illucens* and cause them to produce stress, resulting in the high production of AMPs. Our results indicate that high concentrations (1.22 × 10^9^ and 1.22 × 10^10^ CFU/g) of RP induced AMPs to a stable level. Considering the production costs, we suggest that the optimal concentration of AMPs induced by RP is 1.22 × 10^9^ CFU/g.

#### 4.2.4. Effect of Feed Supplementation with RP on the Intestinal Flora of *H. illucens*

In our study, alpha diversity analysis showed that adding RP to the diet significantly increased the Shannon and Ace diversity indices, which indicates that RP can improve the species richness and evenness of the gut microbiota of *H. illucens*, resulting in a more diverse and evenly distributed microbiota. Moreover, the effect of different concentrations of RP on the composition of the gut flora varied, with higher concentrations of RP having higher flora diversity. Studies have shown that increasing the diversity of the gut microbiome is better for host health [70].

Adding many feed probiotics can influence the structure of the animal’s intestinal flora and improve the host’s health status [71,72]. The results of gut sequencing in this study show that the dominant flora of *H. illucens* is distributed in *Proteobacteria, Firmicutes and Actinobacteria*. *Proteobacteria. Firmicutes* and *Actinobacteria* are most commonly found in insects [73]. In this study, the abundance of *Proteobacteria and Actinobacteria* decreased, and *Firmicutes* increased in *H. illucens* fed with high concentrations of RP, which may be the result of feeding RP. *Proteobacteria* are Gram-negative bacteria, most of which are parthenogenic or exclusively anaerobic [74] and are usually the main component of the insect gut microbiota [73]. A potential disease risk is associated with the increased abundance of *Proteobacteria,* as it includes many pathogenic bacteria. Most *Firmicutes* are Gram-positive bacteria, including many probiotic bacteria such as *Bacillus* and *Lactobacillus*. The high abundance of *Firmicutes* and the low abundance of *Proteobacteria* and *Actinobacteria* in this study suggest that RP improves the microbial composition of the gut.

At the genus level, some common probiotic bacteria, such as *Lactobacillus* and *Rhodopseudomonas*, increased significantly in abundance after feeding RP. *Lactobacillus* and *Rhodopseudomonas* are common probiotics used in animal production farming, and *Lactobacillus* can improve digestibility and immunity in aquatic animals [75]. *Rhodopseudomonas* can promote the growth of probiotics, regulate water quality and enhance animal resistance [76]. With the increased feeding RP concentration, the abundance of *Corynebacterium* and *Proteus* decreased significantly. Except for *Corynebacterium diphtheriae*, *Corynebacterium* is mostly conditionally pathogenic and can cause various clinical infections, such as sepsis, endocarditis and pneumonia, and has attracted the attention of scholars abroad [77,78]. *Proteus* is a genus of conditioned pathogenic bacteria that can cause food poisoning and many diseases under certain conditions [79,80,81]. In this study, feed supplementation with RP increased the abundance of beneficial bacteria and decreased the abundance of harmful bacteria, which may be the result of increased digestibility and disease resistance in *H. illucens*. Bacteria capable of producing multiple extracellular hydrolytic enzymes can be isolated from selective and enriched media in the gut of nutrient-rich larvae [73]. These isolated strains have multiple hydrolytic capabilities that can provide ecological advantages to hosts grown in polymer-rich diets. Specific hydrolytic activity is associated with a specific phylogenetic group [73]. The improved digestibility of *H. illucens* may be related to the composition and abundance of the intestinal flora. The intestinal flora may influence the metabolic function of *H. illucens*, enhancing its digestive capacity and disease resistance. On the one hand, RP influences the nutritional level, material metabolism and immune function of *H. illucens*. Intestinal flora correlates with physiological status, digestive and absorption functions and disease resistance. Thus, RP indirectly influences the nutritional level, material metabolism and immune function of *H. illucens* through the regulation of the intestinal flora. In addition, due to the antibacterial effect of AMPs, the production of AMPs in the body of black soldier fly may inhibit the growth of harmful flora, giving probiotics a better living space and indirectly regulating the balance of gut flora. AMPs are also key to ensuring that the *H. illucens* intestine remains healthy in a putrefactive environment.

## 5. Conclusions

In conclusion, compared to the other strains, RP is more suitable as a feed additive to improve the growth performance, digestibility and induction of AMPs in *H. illucens*. High concentrations (1.22 × 10^9^–1.22 × 10^10^ CFU/g) of RP are more effective in promoting growth and microbial composition of the gut of *H. illucens*, improving digestibility and induction of AMPs.

## Figures and Tables

**Table 1:** Ingredients and nutrient level of experimental diets (based on dry matter).

Ingredients	Content (%)	Proximate Composition	Content (%)
Wheat bran	50	Crude protein	22.68
Soybean meal	30	Crude lipid	3.71
Corn meal	20	Ash	2.38

**Table 2:** Effects of different strains on the growth performance of *H. illucens*.

Parameter	WGR (%)	SGR (%/d)
Control	416.67 ± 47.26 ^c^	32.79 ± 1.80 ^d^
EC	426.67 ± 25.17 ^c^	33.21 ± 0.96 ^cd^
SA	686.67 ± 80.21 ^ab^	41.18 ± 2.03 ^ab^
BS	443.33 ± 20.82 ^c^	33.84 ± 0.77 ^cd^
RP	730.00 ± 43.59 ^a^	42.31 ± 1.04 ^a^
SC	693.33 ± 80.21 ^ab^	41.35 ± 2.04 ^ab^
LP	546.67 ± 51.32 ^bc^	37.29 ± 1.61 ^bc^

Means in the same column with different superscripts are significantly different (*p* < 0.05).

**Table 3:** Effects of different strains on crude protein, crude lipid and ash of *H. illucens*.

Parameter	Crude Protein (%)	Crude Lipid (%)	Ash (%)
Control	53.74 ± 2.08	14.40 ± 2.57 ^b^	2.61 ± 0.10 ^b^
EC	55.13 ± 3.04	14.69 ± 0.52 ^ab^	2.68 ± 0.09 ^ab^
SA	55.36 ± 4.02	15.93 ± 1.18 ^ab^	2.64 ± 0.05 ^b^
BS	58.16 ± 3.72	13.96 ± 0.30 ^b^	2.70 ± 0.10 ^ab^
RP	56.15 ± 3.17	15.59 ± 0.62 ^ab^	2.78 ± 0.12 ^ab^
SC	55.77 ± 3.15	17.90 ± 0.33 ^a^	2.88 ± 0.02 ^a^
LP	56.31 ± 4.11	16.34 ± 1.19 ^ab^	2.76 ± 0.01 ^ab^

Means in the same column with different superscripts are significantly different (*p* < 0.05).

**Table 4:** Effects of different strains on the digestive ability of *H. illucens*.

Parameter	α-Amylase (U/mgprot)	Lipase (U/gprot)	Trypsin (U/gprot)
Control	5.68 ± 1.26 ^cd^	10.04 ± 1.87 ^d^	476.40 ± 62.90 ^c^
EC	3.53 ± 0.85 ^d^	7.39 ± 1.41 ^d^	424.60 ± 59.40 ^c^
SA	6.59 ± 1.29 ^bc^	14.47 ± 2.47 ^bc^	1458.44 ± 274.45 ^b^
BS	6.27 ± 1.09 ^bc^	10.29 ± 0.71 ^d^	1620.73 ± 107.55 ^ab^
RP	12.16 ± 1.75 ^a^	22.07 ± 3.31 ^a^	1869.22 ± 201.88 ^a^
SC	8.41 ± 1.08 ^b^	17.57 ± 2.56 ^b^	1386.38 ± 255.78 ^b^
LP	5.40 ± 1.38 ^cd^	11.03 ± 2.65 ^cd^	712.85 ± 127.94 ^c^

Means in the same column with different superscripts are significantly different (*p* < 0.05).

**Table 5:** Effects of different strains on the concentration of AMPs in *H. illucens*.

Parameter	Antibacterial Peptide (mg/mL)
Control	0.03 ± 0.01 ^c^
EC	0.05 ± 0.01 ^ab^
SA	0.05 ± 0.01 ^b^
BS	0.04 ± 0.01 ^bc^
RP	0.06 ± 0.01 ^a^
SC	0.05 ± 0.01 ^ab^
LP	0.04 ± 0.01 ^bc^

Means in the same column with different superscripts are significantly different (*p* < 0.05).

**Table 6:** The diameter of the inhibition circles of each feed group.

Parameter	Diameter (mm)
NC	0
PC	15.47 ± 0.96 ^a^
Control	8.07 ± 0.91 ^e^
EC	11.50 ± 1.30 ^bcd^
SA	11.53 ± 0.93 ^bc^
BS	9.10 ± 0.92 ^cde^
RP	12.80 ± 0.66 ^b^
SC	11.47 ± 0.95 ^bcd^
LP	8.93 ± 0.47 ^de^

Means in the same column with different superscripts are significantly different (*p* < 0.05).

**Table 7:** Effects of different concentrations of RP on the growth performance of *H. illucens*.

Parameter	WGR (%)	SGR (%/d)
R0	858.00 ± 50.63 ^c^	45.18 ± 1.07 ^c^
R1	992.22 ± 40.05 ^bc^	47.81 ± 0.73 ^bc^
R2	1031.11 ± 40.73 ^b^	48.51 ± 0.73 ^b^
R3	1067.78 ± 103.94 ^ab^	49.10 ± 1.82 ^ab^
R4	1202.44 ± 13.83 ^a^	51.34 ± 0.21 ^a^
R5	1213.33 ± 24.04 ^a^	51.50 ± 0.37 ^a^

Means in the same column with different superscripts are significantly different (*p* < 0.05).

**Table 8:** Effects of different concentrations of RP on crude protein, crude lipid and ash of *H. illucens*.

Parameter	Crude Protein (%)	Crude Lipid (%)	Ash (%)
R0	56.52 ± 2.11	21.39 ± 1.57 ^c^	2.96 ± 0.14
R1	54.71 ± 2.37	23.25 ± 0.56 ^bc^	2.93 ± 0.05
R2	54.10 ± 2.07	24.93 ± 0.46 ^ab^	2.88 ± 0.05
R3	52.88 ± 1.16	25.11 ± 0.60 ^ab^	2.92 ± 0.01
R4	54.05 ± 1.54	26.19 ± 0.56 ^a^	3.22 ± 0.68
R5	53.44 ± 2.78	26.59 ± 0.51 ^a^	2.83 ± 0.04

Means in the same column with different superscripts are significantly different (*p* < 0.05).

**Table 9:** Effects of different concentrations of RP on the digestive ability of *H. illucens*.

Parameter	α-Amylase (U/mgprot)	Lipase (U/gprot)	Trypsin (U/gprot)
R0	5.24 ± 0.46 ^b^	30.54 ± 5.06 ^c^	928.28 ± 39.35 ^c^
R1	5.07 ± 0.75 ^b^	39.32 ± 6.14 ^bc^	1158.97 ± 79.30 ^bc^
R2	7.40 ± 0.76 ^a^	41.07 ± 6.14 ^bc^	1258.97 ± 108.69 ^abc^
R3	6.84 ± 0.79 ^a^	42.53 ± 4.19 ^b^	1224.84 ± 189.44 ^bc^
R4	7.23 ± 0.95 ^a^	48.98 ± 9.57 ^ab^	1559.56 ± 268.55 ^a^
R5	6.84 ± 1.08 ^a^	59.98 ± 7.41 ^a^	1464.06 ± 298.10 ^ab^

Means in the same column with different superscripts are significantly different (*p* < 0.05).

**Table 10:** Effects of different concentrations of RP on the concentration of AMPs in *H. illucens*.

Parameter	Antibacterial Peptide (mg/mL)
R0	0.04 ± 0.01 ^c^
R1	0.07 ± 0.01 ^b^
R2	0.07 ± 0.01 ^b^
R3	0.09 ± 0.02 ^b^
R4	013 ± 0.01 ^a^
R5	0.14 ± 0.02 ^a^

Means in the same column with different superscripts are significantly different (*p* < 0.05).

**Table 11:** The diversity indices of bacteria communities on OTU levels.

Group	Shannon	Simpson	Chao	Ace
Control	1.71 ± 0.11 ^b^	0.36 ± 0.04 ^a^	107.29 ± 5.87 ^a^	107.90 ± 8.21 ^b^
RP-A	1.83 ± 0.08 ^b^	0.34 ± 0.03 ^a^	107.43 ± 6.35 ^a^	109.71 ± 5.22 ^b^
RP-B	2.49 ± 0.23 ^a^	0.16 ± 0.06 ^b^	124.56 ± 9.16 ^a^	135.32 ± 11.39 ^a^

Means in the same row with different superscripts are significantly different (*p* < 0.05).

## Data Availability

The partial data analyzed for this study are available from the corresponding author upon reasonable request.

## Supplementary Materials

The following supporting information can be downloaded at https://www.mdpi.com/article/10.3390/ani13172722/s1. Figure S1: The inhibition circles of each feed group; Table S1: Raw sequencing information of the microbiota composition in the *H. illucens* intestine.
